# Gamma delta T-cell reconstitution after allogeneic HCT: A platform for cell therapy

**DOI:** 10.3389/fimmu.2022.971709

**Published:** 2022-08-29

**Authors:** Ahmed Gaballa, Lucas C. M. Arruda, Michael Uhlin

**Affiliations:** ^1^ Department of Clinical Science, Intervention and Technology, Karolinska Institutet, Stockholm, Sweden; ^2^ Department of Clinical Chemistry, National Liver Institute, Menoufia University, Menoufia, Egypt; ^3^ Department of Immunology and Transfusion Medicine, Karolinska University Hospital, Stockholm, Sweden

**Keywords:** gamma delta, immune reconstitution, immunotherapeutic, hct, allogeneic

## Abstract

Allogeneic Hematopoietic stem cell transplantation (allo-HCT) is a curative platform for several hematological diseases. Despite its therapeutic benefits, the profound immunodeficiency associated with the transplant procedure remains a major challenge that renders patients vulnerable to several complications. Today, It is well established that a rapid and efficient immune reconstitution, particularly of the T cell compartment is pivotal to both a short-term and a long-term favorable outcome. T cells expressing a TCR heterodimer comprised of gamma (γ) and delta (δ) chains have received particular attention in allo-HCT setting, as a large body of evidence has indicated that γδ T cells can exert favorable potent anti-tumor effects without inducing severe graft versus host disease (GVHD). However, despite their potential role in allo-HCT, studies investigating their detailed reconstitution in patients after allo-HCT are scarce. In this review we aim to shed lights on the current literature and understanding of γδ T cell reconstitution kinetics as well as the different transplant-related factors that may influence γδ reconstitution in allo-HCT. Furthermore, we will present data from available reports supporting a role of γδ cells and their subsets in patient outcome. Finally, we discuss the current and future strategies to develop γδ cell-based therapies to exploit the full immunotherapeutic potential of γδ cells in HCT setting.

## Introduction

Over the past 5 decades, allogeneic hematopoietic stem cell transplantation (allo-HCT) has evolved rapidly; over 1 million HCTs have been performed worldwide since the first successful allo-HCT in 1959 (1). This remarkable progress was the result of several factors including the introduction of less toxic conditioning regimens and improved understanding of the immune system (2). Besides being established as an efficient therapeutic option for several hematological and non-hematological disorders, allo-HCT has also served as a platform to develop novel personalized cell therapies (3, 4).

Despite the tremendous improvement in allo-HCT outcome over the past years, several challenges remain, preventing the full therapeutic benefit. Among these challenges, primary disease relapse, graft versus host disease (GVHD), and infectious complications represent the main leading causes of transplant-related morbidity and mortality (5, 6). These complications are linked to the profound immunocompromised state encountered after allo-HCT (7). Therefore, efficient restoration of a functional immune system is central for beneficial outcome (8). In line with this, several studies have indicated that T cells are of key importance in allo-HCT outcome as prolonged lymphopenia after allo-HCT is associated with severe adverse effects (5, 8–10). Proper understanding of T cell reconstitution kinetics and the factors affecting this process following allo-HCT are crucial to improve HCT outcome.

Following allo-HCT, the T cell compartment is restored through a complex and dynamic process that involves two distinct pathways. During the early phase after transplantation T cells recover mainly through the homeostatic proliferation of donor-derived mature T cells co-infused with the stem cell graft (11). This process is also known as the thymic-independent pathway to distinguish it from the thymic-dependent pathway which involves *de novo* generation of naïve T cells from donor progenitor stem cells (12, 13).

Although homeostatic proliferation offers a faster route to replenish the virtually empty T cell pool, the T cells reconstituted in this fashion are relatively inferior to their *de novo* generated T cell counterparts. It has been shown that T cells originating from donor-derived mature T cells offer limited protection against infectious pathogens possibly because of their limited TCR diversity (14–16). Furthermore, they are more prone to activation-induced cell death (17), and can most likely induce GVHD as they are potentially alloreactive (18). On the contrary, *de-novo* generated T cells undergo TCR rearrangement and the stringent selection steps in the thymus resulting in a more diverse repertoire of cells which are self-tolerant (19–21). However, thymopoiesis is a slow and age-dependent process; thus it can take up to several months or years especially in elderly patients. Furthermore, the thymic stroma is extremely sensitive to transplant-related insults such as GVHD, infections, and conditioning regimen (20, 22, 23).

Although T cell reconstitution has been extensively investigated, most studies have focused on the role of conventional αβ T cells while unconventional T cells remain under scrutinized. During the past decades, a subset of T cells known as gamma delta (γδ) T cells has become the focus of increasing interest due to their proven role in stress immunosurveillance (24), as well as their emerging roles in tissue homeostasis, wound healing, and heat regulation (25–29). However, their detailed reconstitution after allo-HCT remains poorly understood. In the next sections we shed light on their main reconstitution features, the factors affecting them, and their role after allo-HCT.

## Immunobiological features of gamma delta (γδ) T cells

Gamma delta (γδ) T cells comprise a distinct lineage of T lymphocytes that can be distinguished from conventional T cells through their TCR that encompasses γ and δ chains instead of the α and β chains (30, 31). Together with T and B lymphocytes, γδ T cells have been conserved across species for millions of years (32). Similar to their αβ T cell counterparts, the development of γδ T cells within the thymus entails somatic rearrangement of the V(D)J segments of their TCR CDR3 region by the recombination activating gene (33). Human γδ TCR is encoded by two distinct gene segments: *TRG* gene segment located on chromosome 7 encoding 6 functional Vγ genes, and the *TRD* gene segment embedded within the *TRA* locus at chromosome 14 encoding 8 functional Vδ genes (31). Based on the Vδ chain usage γδ T cells are classified into two major subsets; the Vδ2+ subset and the non-Vδ2 subset of which Vδ1+ γδ T cells are the predominant fraction (34). In the peripheral blood, 1-10% of total T cells are of γδ T cell lineage, the vast majority of which express the semi-invariant phosphoantigen reactive Vγ9Vδ2 TCR (35, 36). The non-Vδ2 subsets are found predominantly in epithelial tissue compartments such as skin, intestine, and reproductive system and unlike their Vδ2+ counterparts their activating ligands and the underlying recognition mechanisms have, so far, remained incompletely understood (37).

Whether γδ T cells align best with innate or adaptive biology remains undecided. Classically, γδ T cells were described as an interface between innate and adaptive immunity as they share features of both systems (38). Nonetheless, fundamental data from recent studies support distinct innate-like and adaptive-like immunobiological paradigms (39, 40). For instance, several recent studies revealed an antigen-driven clonotypic focusing of certain γδ T cells subsets upon encountering their cognate antigen, alongside transition to an effector memory phenotype supporting adaptive-like paradigm (41–43).

## Receptors and effector functions of γδ T cells

Unlike αβ T cells, the majority of γδ T cells do not express CD4 or CD8 molecules and recognize their antigens in an HLA unrestricted manner (30). Apart from TCR-mediated activation, γδ T cells can be activated in a TCR-independent mechanism (44). Beside toll-like receptors, γδ T cells also express a wide array of NK receptors such as NKG2D, DNAM-1, and the natural cytotoxicity receptors, allowing γδ T cells to react rapidly in response to “altered-self” signals. These features placed γδ T cells in the front line of defense against pathogens and transformed/tumor cells (41, 45).

Upon activation, γδ T cells exert their effector functions either directly or indirectly. The direct cytotoxic effects can be executed through the death receptors (FAS/FAS ligand and TRAIL/TRAIL receptor) triggering target cell apoptosis and/or through perforin/granzyme mediated tumor cell lysis (34, 46). Additionally, γδ T cells express the FcRγIII (CD16) that can mediate direct target cell lysis *via* antibody-dependent cellular cytotoxicity (ADCC) (33, 44). Indirect mechanisms are mediated mainly through cytokines secreted by γδ T cells such as IFN-γ, TNFα, IL-17, and IL-22 (47). These cytokines allow γδ T cells to interact with and modulate the activity of other immune cells. In addition, γδ T cells can promote dendritic cell maturation, take up, process and cross-present antigens to other immune cells, and enhance anti-infectious activities of other immune cells such as NK cells and macrophages (48–50).

## Reconstitution of γδ T cells after allo-HCT: Current understanding

The rate of immune cell recovery after allo-HCT varies from one cell type to another. In general, cells of the innate immune system such as neutrophils, monocytes, and NK cells recover earlier (within weeks), while the recovery of the adaptive immune cells is more prolonged, taking months or even years to entirely recover (51, 52). Only a limited number of studies have focused on the reconstitution of γδ T cells following allo-HCT. In this regard, numerous studies consistently demonstrated that γδ T cell reconstitution occurs in the initial few weeks after transplantation (1, 11, 53–55). In pediatric haplo-identical HCT patients that received αβ T cell-depleted (TCD) grafts, Airoldi et al. showed that γδ T cells expand quickly, reaching up to 90% of the initial T cell pool, and subsequently decline as αβ T cells start to recover. Further characterization of the γδ T cell composition one month post HCT showed that Vδ2 cells were the predominant fraction, indicating that γδ T cell composition did not differ significantly from that present in peripheral blood of the donors or healthy adults (56). Likewise, studying γδ T cell reconstitution at the clonal level showed that reconstituted γδ T cell repertoire remained very stable over time after transplantation (54, 57).

However, it remains unclear whether reconstituted γδ T cells in allo-HCT patients have originated from the peripheral expansion of donor γδ T cells infused within the stem cell grafts or whether they have originated from thymopoiesis. The quick reconstitution of γδ T cells shortly after allo-HCT suggests a peripheral expansion of donor-derived γδ T cells. Corroborating this, γδ T cell reconstitution has been shown to be delayed in patients that received OKT3 TCD grafts (58). Furthermore, by examining the CDR3 size distribution pattern in 23 allo-HCT recipients, Hirokawa et al. identified 2 γδ T cell clones in the donor grafts that were found as well in the recipient blood after allo-HCT, but not before (54). Consistently, chimerism analysis in the first 2-3 weeks after haplo-identical HCT indicated donor-origin of the reconstituted γδ T cells (56). Altogether, these data strongly support that initial γδ T cell reconstitution occurs mainly through the homeostatic peripheral expansion of donor-derived mature γδ T cells.

On the other hand, it has been shown that the proportion of naïve γδ T cells increases as early as 2-3 months after HCT (59). In a recent study, Raven et al. used high throughput RNA-based next generation sequencing to in-depth evaluate the TCR repertoires of 6 allo-HCT patients and their corresponding donors. Their results indicated the presence of heterogenous overlapping sequences in donor/recipient pairs, yet most of patient/donor pairs displayed no specific correlation of their repertoires, supporting *de novo* generation of γδ T cells (57). Altogether, these data suggest that γδ T cell reconstitution involves both thymic-dependent and independent mechanisms, even though γδ T cells rely principally on homeostatic proliferation during the early phase post allo-HCT.

Some questions remain which warrant further investigation. To what extent does the thymus contribute to γδ T cell reconstitution especially in long-term survivors and do *de-novo* generated γδ T cells provide a more favorable patient outcome?

## Factors affecting γδ T cell reconstitution

Unlike αβ T cells, the impact of different transplant-related factors, such as graft source, GVHD, conditioning regiment etc. on γδ T cell reconstitution and TCR diversity remains largely unknown (**Table 1**). In a recent retrospective study of 202 adult AML patients, Klyuchnikov and colleagues identified several transplant-related factors associated with γδ T cell reconstitution after allo-HCT (60). Their results indicated that younger recipient/donor age, sex mismatch, use of a matched donor, and CMV reactivation were factors associated with faster γδ T cell reconstitution. Additionally, they showed that the use of post-transplantation cyclophosphamide was associated with lower levels of γδ T cells compared to ATG (60). Of note, the impact of cytomegalovirus (CMV) infection/reactivation on γδ T cell reconstitution and TCR repertoire after allo-HCT has been discussed thoroughly in multiple studies including a recent review by our group (68).

**Table 1:** Factors affecting γδ T cell reconstitution.

Study	Study Cohort	Factors examined	Impact on γδ
Klyuchnikov et al. (60)	Adult AML patients (n = 202) Median follow-up (28 months)	Younger recipient/donor age	↑
		Sex mismatch	↑
		use of a matched donor	↑
		CMV reactivation	↑
		The use of ATG	↑
Cela et al. (61)	31 recipients of TCD BMT	GVHD	No
		Infection (viral/fungal)	↑
Perko et al. (62)	Retrospective study including 102 Pediatric ALL/AML patients Mean follow-up (2.7 years)	donor type (MRD vs others)	↑
		gender	No
		diagnosis	No
		GVHD	No
		CD3 number	↑
de Witte et al. (55)	MSD/MUD cohort (n= 28) and UCB (n=26)	UCB vs other	↓
		CMV infection	↑ (vδ2- T cells)
Eyrich et al. (63)	Prospective study of 25 pediatric patients; 13 received CD34+ selected PBSC from unrelated donors, 12 received unmanipulated BM from matched siblingsMedian follow-up 1157 days	PBSC recipient’s vs unmanipulated BM recipients	↓
Lamb et al. (58)	43 patients received *ex vivo* T cell depletion using T10B9 mAb (αβ depletion) and 100 patients received TCD grafts using OKT3 mAb (anti-CD3)	αβ TCD grafts vs OKT3 TCD grafts	↑
Airoldi et al. (56)	Prospective study including 27 allo-HCT pediatric patients that received αβ TCD grafts from haplo-identical donors. compared to 9 children that received CD34+ enriched grafts	αβ TCD grafts vs CD34+ enriched grafts	↑
Keever et al. (64)	A total of 195 that received either unmanipulated BM grafts (n=100), αβ TCD grafts (n=67), and elutriated grafts (n=28)	unmanipulated BM grafts vs *ex-vivo* αβ TCD grafts	No diff.
Otto et al. (65)	PBMC obtained from 6 donors after G-CSF	G-CSF	γδ T cells retained their effector function
Bian et al. (66)	20 donors before and after G-CSF mobilization	G-CSF	no change in proportions or functions
Minculescu et al. (67)	49 donors before and after G-CSF mobilization	G-CSF	↑ naïve and terminally differentiated effector (TEMRA) γδ T cells and ↓ memory cells

↑, increase; ↓, decrease.

Whether graft source impacts the rate of γδ T reconstitution after allo-HCT has only been adequately investigated in a handful of publications (**Table 1**). Perko et al. examined different variables associated with γδ T cells reconstitution post allo-HCT in 102 pediatric patients with acute leukemia. Their results indicated significant impact of donor source on γδ T cells reconstitution (62). Another study by De witte et al. showed that γδ T cells were significantly fewer in umbilical cord blood recipients compared to recipients of HLA matched siblings or unrelated donors (55). Furthermore, in allo-HCT pediatric patients, the recovery of γδ T cells was delayed in recipients of CD34+ selected PBSC compared to recipients of unmanipulated BM grafts from HLA identical siblings (63, 69). However, it is difficult to conclude whether this is the effect of the graft source per se or due to the manipulation of PBSC graft.

To further examine the impact of graft manipulation on immune reconstitution after HCT, Lamb et al. assessed γδ T cell reconstitution after *ex vivo* T cell depletion using T10B9 mAb (αβ depletion) and OKT3 mAb (anti-CD3). They showed better γδ T cell reconstitution in patients that received αβ TCD grafts (58). In line with this, Airoldi et al. showed that γδ T cells were significantly higher in haplo-identical allo-HCT pediatric patients that received αβ TCD grafts compared to patients that received CD34+ enriched grafts (56). Conversely, results by Keever-Taylor et al. indicated no differences in γδ T cell recovery between allo-HCT patients that received unmanipulated BM grafts or αβ TCD grafts, although they showed that the reconstitution of NK cells was faster in the TCD group (64).

The effect of stem cell mobilization using granulocyte colony stimulating factor (G-CSF) on γδ T cells has been scarcely investigated. Otto et al. showed that γδ T cell effector functions were not impaired in G-CSF mobilized stem cell grafts (65). In line with this, Bian et al. examined phenotypic characteristics of γδ T cells in the peripheral blood of 20 donors before and after G-CSF mobilization. They showed that γδ T cells retained their homeostatic proportions and IFN-γ secreting capabilities following G-CSF (66). Conversely, a more detailed analysis by Minculescu et al. showed that G-CSF preferentially mobilized naïve and terminally differentiated effector (TEMRA) γδ T cells over memory cells with a preferential increase in the non-Vδ2 subset while also increasing the proportion of HLA-DR expressing γδ T cells (67). Regarding the impact on TCR repertoire, it has been shown that G-CSF was associated with TCR repertoire disturbances in the form of alteration of distribution and clonality of some *TRG* and *TRD* subfamilies, suggesting a potential immune modulatory effect (70). Further investigations are required to elucidate the detailed impact of G-CSF mobilization on different γδ T cell subsets.

## γδ T cell reconstitution and clinical outcome after allo-HCT

Multiple studies have highlighted the importance of conventional αβ T cell recovery and its impact on clinical outcome post allo-HCT, but the role of γδ T cells has not been well described. Whether γδ T cells are beneficial or not especially in the context of GVHD has been a matter of debate, particularly among earlier studies that showed controversial results. For instance, Viale et al. showed that percentages and total numbers of γδ T cells were increased in patients that developed acute GVHD up to 3 months after allo-HCT (71). Likewise, other studies in mice have suggested a role for γδ T cells in GVHD pathogenesis (72–74). For instance, using an experimental GVHD murine model, Maeda et al, showed that host γδ T cells exacerbate GVHD by enhancing the alloreactive capacity of DCs in a cell contact dependent manner (74). However, several lines of evidence from both murine and human studies support that γδ T cells are not involved in the initiation or severity of GVHD (44, 53, 75, 76). In their study, Drobyski et al. found that lethally irradiated mice that were infused with large doses of γδ T cells did not develop GVHD (75). Likewise, similar findings in human studies were reported. Cela et al. didn’t find any significant correlation between γδ T cells and the incidence of GVHD in the first 12 months posttransplant (61). Corroborating this, Lamb et al. showed that donor-derived γδ T cells were able to recognize and lyse primary ALL blasts but did not proliferate when cultured with allogeneic cells in a mixed lymphocyte reaction, suggesting a graft vs leukemia (GVL) activity in the absence of an allogeneic response (76). Additionally, several studies showed decreased incidence of GVHD in recipients of αβ TCD grafts.

In the same context, the impact of GVHD on γδ T cell reconstitution has not been adequately addressed after allo-HCT. Although previous studies have shown that GVHD and/or its treatment can impair thymic function and thus T cell recovery (77, 78), it remains unclear to what extent γδ T cell reconstitution is affected by GVHD. In an earlier study that included 31 recipients of TCD BM grafts no significant impact of GVHD on the pattern of γδ T cell recovery was found (61). Of note, in-depth analysis of γδ T cell reconstitution might reveal a potential impact of GVHD on γδ T cells at functional or clonal level.

It was not long before the anti-tumorous capabilities of γδ T cells were confirmed in human studies after it was first reported in an experimental mice model (79). In fact, the ability of γδ T cells to exhibit potent anti-tumor responses in the absence of the unwanted alloreactive immune response as well as their potential to bypass tumor immune evasion mechanisms (e.g. HLA down regulation) have made γδ T cells a subject of great interest in the field of allo-HCT (80). Several research groups have shown that elevated γδ T cells early post-transplantation was associated with improved leukemia-free survival and overall survival (81, 82). In fact, the earliest clinical report that suggested favorable γδ T cell role post allo-HCT, in terms of enhanced GVL effect and favorable survival outcome, was published in 1996 by Lamb et al. (81). In this study that included 43 leukemia patients that received αβ TCD grafts from partially mismatched related donors, the authors showed that disease-free survival was significantly improved in patients that developed high proportion (> 10%) of γδ T cells during the first 6 months after transplantation compared to patients with normal γδ T cell proportion. In a subsequent follow up (3 years) of this study, the authors confirmed the previous results and further showed that Vδ 1 cells were the predominant γδ T cell population in patients with high γδ T cell proportion (58). In an extension of this study with additional patients and longer follow up (up to 8 years), Godder at al. confirmed the long-term survival advantage with no increased incidence of acute GVHD in patients with higher numbers of γδ T cells (82). Likewise, Perko et al. reported decreased incidence of infection and improved event-free survival in patients with elevated γδ T cells (62). Corroborating these findings in pediatric setting, a long-term prospective study of children with acute leukemia that underwent αβ T- and B-cell–depleted haplo-HCT after a myeloablative regimen showed decreased incidence of both acute and chronic GVHD as well as, reduced incidence of none relapse mortality, conceivably due to the spared γδ T cells and NK cells transferred with the graft (83). Similarly, in pediatric haplo-identical HCTs with αβ TCD grafts, Park et al. showed improved relapse-free survival in acute leukemia patients that recovered with high percentage of γδ T cells at day 30 compared to those with low percentage of γδ T cells (84).

In the context of a potential pathogen protective role, it has been confirmed in multiple studies that CMV reactivation induces clonal expansion of non-Vδ2+ T cells (85, 86). Likewise, several studies have highlighted the role of Vδ1 T cells in CMV immunosurveillance after allo-HCT (85, 87). Furthermore, a more recent study in pediatric patients indicated that patients with a low percentage (≤21%) of γδ T cells at day 30 had significantly higher incidence of CMV reactivation compared to patients with a high percentage (84). Additionally, a possible role for γδ T cells in EBV immune response was suggested by De Paoli et al. (88). In a recent systematic review and meta-analysis we addressed whether γδ T cell reconstitution is associated with clinical outcome after allo-HCT. Out of 2412 studies, 11 studies (919 patients, median follow-up of 30 months) met the eligibility criteria for the meta-analysis. Results of the meta-analysis confirmed the benefit of having higher levels of γδ T-cells in peripheral blood after HCT in terms of less disease relapse, fewer viral infections, and improved overall and disease-free survivals, whereas there was no association with the incidence of acute GVHD (89).

Given that donor γδ T cells co-infused within the stem cell (SC) grafts substantially contribute to the reconstituted γδ T cell pool after allo-HCT, investigating γδ T cells graft composition can provide insight into clinical outcome. In this context, an earlier study showed increased cumulative incidence of acute GVHD II-IV in patients that received PBSC grafts with higher γδ T cells content (90). However, in this study γδ T cells were examined in total and the impact of different subsets was not investigated. Given that γδ T cells comprise heterogenous subsets, we recently analyzed the composition of γδ cell subsets in 105 donor stem cell grafts. We found that patients that received SC grafts containing higher proportions of CD8+γδ T cells had an increased cumulative incidence of acute GVHD II-III, suggesting a potential alloreactive role of this subset (91). In the same study, we found an inverse correlation between CD27+ γδ T cell graft content and the incidence of both relapse and CMV reactivation post allo-HCT (91). Though we have not assessed the functional properties of this subset, previous studies on murine γδ T cells have shown that CD27 demarcates IFNγ producing γδ cells (92). Altogether, these data suggest that the composition of intra-graft γδ T cell subsets can impact patient outcome after allo-HCT.

In addition to γδ T cell proportion, γδ TCR repertoire has been suggested to play a role in clinical outcome as it has been shown that γδ T cells expressing polyclonal TCR repertoire comprise different affinities and hence different functional capabilities (46). In line with this assumption, Grunder et al. showed that Vγ9Vδ2 T cells with a polyclonal TCR repertoire are inferior to cells that express a monoclonal repertoire (93). In a recent study, we examined the impact of intra-graft γδ T cell repertoire composition on the outcome of 20 adult AML patients that underwent allo-HCT. Analysis of the *TCR*γ repertoire by NGS indicated that grafts given to non-relapsed patients featured a more public repertoire and an increased presence of long sequence clonotypes. Further analysis of the amino acid sequences showed that 12 public and 4 private sequences were exclusively found in high frequencies in grafts given to non-relapsed patients most of these sequences were 42-54 amino acid long (94). Altogether, these data suggest that specific γδ TCR clonotypes might play important role in patient outcome after transplantation, adding another layer of complexity to the already intricate landscape.

## Concluding remarks and future directions

In general, most of the ongoing and future directions to harness γδ T cells in allo-HCT fall under two main categories: strategies that aim to enhance γδ T cell reconstitution, and strategies that aim to exploit their anti-tumor properties by redirecting their immune responses against tumors. We briefly highlight some of these approaches. For more comprehensive information the readers are directed to these reviews (30, 46, 48, 95, 96).

The notion that γδ T cell-enriched grafts are associated with better γδ T cell reconstitution and more favorable outcome has encouraged researchers to develop strategies that enhance γδ T cell recovery post-HCT. Beside the use of αβ TCD grafts, another strategy that has been investigated is the adoptive transfer of autologous or allogeneic γδ T cells. In this strategy γδ T cells are enriched and re-infused either directly or after *in vitro* expansion. An example of such strategy is the use of αβ TCD donor lymphocyte infusion. This strategy would have the benefit of retaining higher numbers of γδ T and NK cells without the unfavorable alloreactive donor T cells. Our group has investigated the use of αβ TCD grafts as post-transplant boosters to treat secondary graft failure in 5 allo-HCT patients. The results were promising as there was no signs of GVHD or other side effects (97). A subsequent follow up of this study with more patients and longer follow up time supported the same conclusion (98).

Accumulating evidence indicates that the thymus is, to a certain extent, implicated in the reconstitution of γδ T cells after allo-HCT (57). Therefore, it is reasonable to assume that enhancing thymic regenerative capacity would positively improve γδ T cell recovery. Even though the clinical benefit of γδ T cells reconstituted through the thymic-dependent pathway is still unknown, enhancing the thymic function would still be beneficial for conventional T cell reconstitution. There are several reviews that have discussed the different strategies that are investigated to enhance thymic regenerative capacity, and the reader is referred to some publications for further information (20, 99, 100).

Since the first observation of the ability of aminobisphosphonate to selectively expand Vδ2Vγ9 T cells in multiple myeloma patients (101), several clinical trials have been conducted in solid and hematological cancers using zoledronate (ZOL) and low dose IL-2 for either *in vivo* or *ex vivo* expansion of Vδ2Vγ9 cells (30, 49). Although the use of Zol/IL-2 in these trials proved to be safe and tolerable, the resulting outcome was limited. This could, in part, be due to the functional plasticity, the infusion of polyclonal TCR repertoire, and/or cell exhaustion, emphasizing the need for further optimization of current protocols. In this regard, several strategies have been suggested to enhance the proliferative and functional capacity of *ex-vivo* expanded γδ T cells. For instance, a recent study showed that γδ T cell expansion was more efficient when a bisphosphonate prodrug was used. Furthermore, several studies have investigated the role of common γ-chain family cytokines such as IL-15. Results showed that γδ T cells expanded in presence of IL-15 displayed enhanced cytotoxic capabilities (102), and upregulated CD56, a marker associated with better cytotoxic effector function (103). Although TGF-β is generally regarded as an immunoregulatory cytokine, recent reports showed enhanced cytotoxicity and IL-9 secretion in TGF-β-treated, phosphoantigen activated γδ T cells (103). Besides, the administration of vit C during γδ T cell expansion has been recently explored (104).

Unlike Vδ2Vγ9 cells, protocols for Vδ1+ expansion have not been explored until recently due to the lack of specific agonist for Vδ1+ cells. So far, only a limited number of large-scale protocols for Vδ1+ expansion have been described (105, 106). In this regard, Wu et al. developed a protocol for preferential expansion of Vδ1+cells from PB of healthy donors and colon cancer patients using phytohemagglutinin (PHA) and IL7. To develop a GMP-compatible protocol that can be clinically adapted, Almeida et al. described a 3 week expansion protocol known as delta one T cell (DOT). Unlike previous protocols they did not use mitogenic stimulators like PHA, instead they used TCR stimulation and a defined cytokine cocktail. This protocol resulted in up to 2000-fold expansion of Vδ1+ cells that displayed antitumor capabilities with no IL17 production (107).

The redirection of T-cell responses against specific tumor antigens represents one of the mainstays of modern personalized precision medicine. Although the use of γδ T cells has lagged behind αβ T cells in this field, they offer an attractive alternative due to their rapid innate like response and lower alloreactivity. In this regard, engineering γδ T cells to express a chimeric antigen receptor (CAR) is currently under clinical development. In fact, γδ T cells that express a first-generation CAR directed against GD2 were first described in 2004 (108). Another interesting approach is the use of T cells engineered with defined γδ TCRs (TEG). Unlike the CAR γδ T cells, in this strategy αβ T cells are transduced to express a high-affinity Vγ9Vδ2 TCR providing features of both conventional and unconventional immune cells (109). A phase I trial is currently ongoing to test the safety of TEG in patients with a relapsed/refractory AML, high-risk Myelodysplastic Syndrome (MDS) or relapsed/refractory Multiple Myeloma (MM) (NTR 6541). Finally, redirecting γδ T cell against tumor antigens can be achieved using bispecific antibodies. These are nano constructs that comprise 2 single chain (sc) Fv domains where one scFv binds to the effector and the other binds its target (e.g. tumor antigen). In this regard, [HER2 ×Vγ9] bispecific T cell engager (BiTE) has been developed and tested by Oberg et al. (110). The same group have also developed and tested a Tribody [(HER2)2 × CD16] that redirect CD16-expressing γδ and NK cells against HER2-expressing cancer cells. The new tribody was shown to be effective in enhancing γδ T cell and natural killer cell cytotoxicity (111).

In conclusion, the inevitable favorable role of γδ T cells in allo-HCT setting has stimulated researchers to exploit their full immunotherapeutic benefits. However, as we discussed above, caution should be paid as to which subset and what function it may exert. Therefore, a better understanding of the functional heterogeneity of the γδ T cell compartment, mechanisms of antigen recognition, and γδ TCR ligands are fundamental to exploit the full therapeutic benefit of γδ T cells.

## Author contributions

AG, LA, and MU designed the study. AG drafted the manuscript. LA and MU reviewed and approved the final manuscript.

## Acknowledgments

This work was funded by grants from the Swedish Cancer Society, the Swedish Childhood Cancer Fund, the Swedish Research Council, and by grants provided by Stockholm County Council.

## Conflict of interest

The authors declare that the research was conducted in the absence of any commercial or financial relationships that could be construed as a potential conflict of interest.

## Publisher’s note

All claims expressed in this article are solely those of the authors and do not necessarily represent those of their affiliated organizations, or those of the publisher, the editors and the reviewers. Any product that may be evaluated in this article, or claim that may be made by its manufacturer, is not guaranteed or endorsed by the publisher.
